# Few-Shot Graph Anomaly Detection via Dual-Level Knowledge Distillation

**DOI:** 10.3390/e27010028

**Published:** 2025-01-01

**Authors:** Xuan Li, Dejie Cheng, Luheng Zhang, Chengfang Zhang, Ziliang Feng

**Affiliations:** National Key Laboratory of Fundamental Science on Synthetic Vision, Sichuan University, Chengdu 610065, China; lixuanlmw@stu.scu.edu.cn (X.L.); zhangluheng@sptc.edu.cn (L.Z.); chengfangzhang@scpolicec.edu.cn (C.Z.); fengziliang@scu.edu.cn (Z.F.)

**Keywords:** anomaly detection, graph neural network, cross entropy, knowledge distillation

## Abstract

Graph anomaly detection is crucial in many high-impact applications across diverse fields. In anomaly detection tasks, collecting plenty of annotated data tends to be costly and laborious. As a result, few-shot learning has been explored to address the issue by requiring only a few labeled samples to achieve good performance. However, conventional few-shot models may not fully exploit the information within auxiliary sets, leading to suboptimal performance. To tackle these limitations, we propose a dual-level knowledge distillation-based approach for graph anomaly detection, DualKD, which leverages two distinct distillation losses to improve generalization capabilities. In our approach, we initially train a teacher model to generate prediction distributions as soft labels, capturing the entropy of uncertainty in the data. These soft labels are then employed to construct the corresponding loss for training a student model, which can capture more detailed node features. In addition, we introduce two representation distillation losses—short and long representation distillation—to effectively transfer knowledge from the auxiliary set to the target set. Comprehensive experiments conducted on four datasets verify that DualKD remarkably outperforms the advanced baselines, highlighting its effectiveness in enhancing identification performance.

## 1. Introduction

Anomaly detection in graphs intends to identify nodes that exhibit abnormal behaviors, significantly deviating from the majority of nodes [1]. This task has numerous high-impact applications across various domains, including detecting abnormal users [2,3] and identifying fraudulent behavior [4,5]. To effectively identify anomalies within graph-structured data, it is essential to develop robust classification models with high generalization capabilities. To achieve this, various techniques such as graph neural networks (GNNs) and matrix factorization have been explored for anomaly detection [6,7].

For graph anomaly detection tasks, obtaining label information is generally costly and time-consuming [1]. Therefore, existing methods are predominately developed in an unsupervised manner. However, the anomalies they identify may turn out to be data noises or uninteresting data instances due to the lack of prior knowledge on the anomalies of interest. Hence, leveraging several labeled samples as the prior information to improve anomaly detection has become a trend, since it is relatively low-cost in real-world scenarios to collect a few labels. Correspondingly, few-shot learning has been introduced to train machine learning models, such as graph convolutional networks (GCNs) [8], on datasets with a limited number of labeled nodes for tasks like anomaly detection and node classification.

Previous few-shot methods may be divided in two primary methodologies: meta-learning-based and self-training-based approaches [9,10]. Meta-learning approaches aim to train an initialization module using prior information from an auxiliary set, employing techniques such as prototypical networks. For example, G-META [9] utilizes meta-learning to maintain the structural and feature knowledge of graphs, while GPN [11] leverages prototypical networks to compute prototypes that capture expressive node representations. Despite their effectiveness, meta-learning methods require the construction of multiple tasks to achieve generalization, which can be time-consuming. Furthermore, these methods often overlook the valuable information contained in unlabeled nodes, limiting their overall effectiveness.

To this end, self-training methods have been designed to utilize prior information from the auxiliary data with low time overhead while assigning pseudo-labels to a portion of the unannotated data of the target set. For example, IA-FSNC [12] cuts down time costs through building a single GCN based on the auxiliary set and allocates pseudo-labels to unlabeled examples with small information entropy in the target set to achieve information augmentation. However, some limitations need to be addressed. For instance, self-training few-shot methods often fail to thoroughly exploit the knowledge contained in the auxiliary set. For IA-FSNC, solely the parameters from the initial layer of the graph convolution are migrated to initialize the target set, overlooking information from other graph convolutional layers. This may lead to an insufficient transfer of information, resulting in a lack of comprehensive knowledge for the target set.

In this paper, we introduce a dual-level knowledge distillation-based graph anomaly detection approach, DualKD, which can significantly enhance the detection performance in scenarios with limited labeled samples. Our approach begins by training a teacher model to produce prediction distributions, which serve as the soft labels of samples to capture entropy-related uncertainties. Subsequently, a student network is trained to learn more detailed node features from these soft labels. The student model is then utilized for the final generalized few-shot anomaly detection, leveraging the enriched knowledge transferred from the teacher model. This knowledge transfer enables the distillation of information from auxiliary datasets, enhancing the detection accuracy on the target dataset while requiring only a few labeled samples.

To maximize the transfer of information from the auxiliary set generated by the teacher model, we devise a dual-level representation distillation mechanism that conveys data from every layer of graph convolution in the teacher model to the student model. This representation distillation consists of two processes: short representation distillation and long representation distillation. To advocate the robustness, the representation distillation loss is combined with the teacher–student distillation loss to form the final distillation loss.

Our contributions are summarized as follows:

- We introduce a dual-level knowledge distillation-based graph anomaly detection framework, DualKD, which can effectively handle scenarios with limited labeled samples.
- We design a dual-level representation distillation strategy that incorporates both short- and long-representation distillation processes to enhance the model’s generalization capabilities.
- We provide experimental evidence for four datasets demonstrating that DualKD outperforms state-of-the-art approaches.

## 2. Related Work

This work is mainly related to three research areas: graph anomaly detection, knowledge distillation, and few-shot learning. Here, we present an overview of the most closely related works in each area.

### 2.1. Anomaly Detection on Graphs

Since graph-structured data are ubiquitous and have the capacity to model a wide range of real-world complex systems, identifying anomalies in graphs has drawn increased research interest [13,14]. Due to their demonstrated superior modeling power for graphs, various GNN-based methods have been proposed to detect anomalies on graphs. The pioneer used GNNs to build an autoencoder to simultaneously reconstruct the attribute and structure information, and the abnormality is evaluated by reconstruction errors [15]. Based on this framework, a tailored deep GCNN is designed to detect local, global, and structural anomalies by capturing community structure in the graph [16]. Contrastive learning and self-supervised learning [17,18] are also introduced to identify the anomalies in attributed networks [19,20]. Meta-learning and hypersphere learning are incorporated into GNNs to leverage the labeled samples for anomaly detection [21,22,23,24].

To remedy the problem that numerous neighbors with normal labels might make the anomaly representations learned by GNNs less distinguishable, multiple re-sampling (e.g., oversampling and undersampling) strategies are designed in [25,26,27]. Researchers also utilized re-weighting methods to assign different weights to different samples [28,29,30]. More recently, spectral filters and counterfactuals are explored to enhance the expressive power of GNNs for learning better anomaly representations [31,32,33,34].

### 2.2. Knowledge Distillation

Knowledge distillation [35] initially emerged for model compression, aiming to guide a comparatively simple student model using a well-trained teacher model characterized by a more complex structure and a greater number of parameters. Building on this, several knowledge distillation methods have been proposed for graph neural networks (GNNs). For instance, G-CRD [36] presents a novel graph contrastive representation distillation for GNNs, employing contrastive learning to align student node embeddings with teacher node embeddings in a shared representation space. GFKD [37] designs a method for knowledge distillation with GNNs that does not involve any training data. Meanwhile, GLNN [38] introduces a high-accuracy, low-delay distillation model by using the teacher model as a GNN and the student model as a multi-layer perceptron. GraphKD [39] and KD-FSNC [40] explore knowledge distillation for graph node classification, which distill auxiliary data to the student for enhancing classification performance on the target data.

### 2.3. Few-Shot Learning

Few-shot learning is a paradigm within deep learning designed to address the challenges associated with training models when there is only a limited amount of labeled data available for each class. Few-shot methods can be divided into two types, i.e., data-based methods and model-based methods. Data-based methods involve learning an augmentation mapping, which maps the training data to new data, and then utilizes the newly generated data to expand the training set in few-shot tasks. For example, FewGAN [41] uses generative adversarial networks to create additional samples. FEFS [42] proposes a data augmentation method based on the assumption that each data dimension is modeled by a Gaussian distribution, where categories that are alike share similar distribution characteristics. The mean and variance of the novel (target) set are adapted based on those from the base (auxiliary) set. On the other hand, model-based methods address the few-shot learning problem by constraining the size of the hypothesis space. For instance, TRPN [43] utilizes intra-class commonality and interclass uniqueness between support samples to estimate the relationship and adjacency relationship between different support–query pairs.

*Difference*: Compared to the aforementioned works, our approach focuses on few-shot anomaly detection on graphs using knowledge distillation. To enhance detection performance, we propose a novel distillation framework that combines soft and hard target losses as the final objective for the student model. In addition, we introduce a dual-level knowledge transfer strategy to effectively capture information from multiple layers. Furthermore, we employ a graph attention network as the backbone architecture for both the teacher and student models.

## 3. Methodology

**Figure 1:**
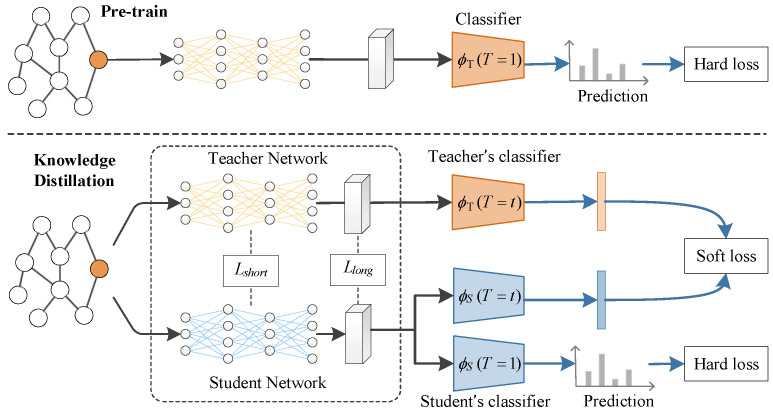
Overall framework of DualKD.

This section gives the formal expression of the proposed method and explains the functions and working mechanism of each part in the expression. As depicted in Figure 1, DualKD encompasses three key elements: a pre-trained GNN module that serves as the backbone for both teacher and student, a soft label distillation process that conveys a class relationship from the teacher to the student, and a representation distillation process that transfers information from every layer of graph convolution in the teacher to the student for learning.

### 3.1. Pre-Training

Inspired by the advances on graph learning [44,45], we introduce the graph attention networks (GATs) [46] as the backbone, which introduces the masked attention mechanism to represent the importance of different adjacent nodes. Formally, in each layer l−1, node $v_{i}$ integrates the features of neighboring nodes to obtain representations of layer *l* via:

(1) $$\begin{array}{c} h_{i}^{\left(l\right)}=\sigma \left(\sum_{j\in V_{i}\cup \left\{v_{i}\right\}}a_{ij}W\cdot h_{j}^{\left(l-1\right)}\right), \end{array}$$

where σ refers to a nonlinear activation function (e.g., ReLU), $V_{i}$ is the set of neighbors for $v_{i}$, and $a_{ij}$ represents the attention coefficient between node $v_{i}$ and node $v_{j}$, which can be computed as:

(2) $$\begin{array}{c} a_{ij}=\frac{\exp(\sigma \left(a^{T}\left[Wh_{i}^{\left(l\right)}⊕Wh_{j}^{\left(l\right)}\right]\right))}{\sum_{k\in V_{i}\cup V_{i}^{\prime }\cup \left\{v_{i}\right\}}\exp\left(\sigma \left(a^{T}\left[Wh_{i}^{\left(l\right)}⊕Wh_{k}^{\left(l\right)}\right]\right)\right)}, \end{array}$$

where ⊕ is the concatenation operation and attention vector *a* is a trainable weight vector that assigns importance to different neighbors of node $v_{i}$, allowing the model to highlight the features of the important neighboring node that is more task-relevant.

To incorporate a high-order neighborhood, multiple layers are adopted to build the graph attentive encoder:

(3) $$\begin{array}{cc} h_{i}^{\left(1\right)} & =\sigma \left(\sum_{j\in V_{i}\cup V_{i}^{\prime }\cup \left\{v_{i}\right\}}a_{ij}^{\left(1\right)}W^{\left(1\right)}\cdot x_{j}\right),\\ & \cdots \cdots \\ z_{i} & =\sigma \left(\sum_{j\in V_{i}\cup V_{i}^{\prime }\cup \left\{v_{i}\right\}}a_{ij}^{\left(L\right)}W^{\left(L\right)}\cdot h_{j}^{\left(l-1\right)}\right), \end{array}$$

where $z_{i}$ is the latent representation of node $v_{i}$. In this way, the graph attentive encoder is able to map the learned node representations by capturing the nonlinearity of topological structure and node attributes.

Next, the MLP with a Sigmoid function is adopted to detect anomalies. The aggregated representations $z_{i}$ are then fed into another MLP with a Sigmoid function to compute the abnormal probability $p_{i}$. The weighted cross-entropy loss is then used for the model training:

(4) $$\begin{array}{c} L=\sum_{i}\left(\varphi y_{i}\log\left(p_{i}\right)+\left(1-y_{i}\right)\log\left(1-p_{i}\right)\right), \end{array}$$

where φ is the proportion of anomaly labels ($y_{i}=1$) to normal labels ($y_{i}=0$).

### 3.2. Soft Label Distillation

Once the GAT is trained using the auxiliary data, we clone the trained model and transfer it to teacher model $\phi_{t}$ and student model $\phi_{s}$

**Teacher Model**: The teacher model is designed to extract comprehensive structural insights from the input data and deliver precise predictions. Thanks to its extensive training and high capacity, the teacher model effectively generalizes, setting a benchmark for developing a more resilient student model [39]. Specifically, the teacher model is utilized to produce soft targets, which are probability distributions across classes, generated by applying a high temperature to the softmax function. Unlike hard labels, these soft targets provide richer information, revealing the relative likelihoods among different classes. The teacher model typically uses a cross-entropy loss, which strives to maximize likelihoods belonging to the correct class. This approach fine-tunes the model parameters to align the predicted probabilities as closely as possible with the actual labels. The objective of the teacher model can be represented:

(5) $$\begin{array}{c} L_{\mathrm{teacher}}=-\sum_{i}y_{i}\log\left(p_{i}\right), \end{array}$$

where $y_{i}$ stands for the real annotation; $p_{i}$ describes the inferred likelihood of category *i*. By leveraging the graph attention network and temperature scaling, the teacher model can produce well-calibrated soft labels that accurately reflect the underlying distribution of anomalies.

**Student model**: The student model aims to maintain high prediction accuracy, even when trained on a more limited dataset. By leveraging the soft targets provided by the teacher, the student model can advocate its generalization and more efficiently consider the inherent characteristics within the dataset. Training the student includes adopting a combination of two losses: the cross-entropy loss regarding soft targets as well as the cross-entropy loss regarding hard targets. For the former, the objective is to align the student forecasts with the teacher soft labels using the cross-entropy loss of the soft labels and the predicted probabilities of the student. This specific loss function is formulated as:

(6) $$\begin{array}{c} L_{\mathrm{soft}}=-\sum_{i}p_{i}\log\left(q_{i}\right), \end{array}$$

where $p_{i}$ denotes the soft target probability generated by the teacher model, while $q_{i}$ represents the predicted probability from the student model, both calculated at an elevated temperature setting.

Aside from learning from soft labels, the student is also learned to predict the actual hard class labels. The cross-entropy loss with hard targets employs the standard softmax function (temperature set to 1) to calculate the loss between the true labels and forecasted probabilities. This loss is defined as:

(7) $$\begin{array}{c} L_{\mathrm{hard}}=-\sum_{i}y_{i}\log\left(q_{i}\right), \end{array}$$

where $y_{i}$ stands for the real annotation. $q_{i}$ represents the inferred probabilities from the student model at a normal temperature.

As a result, the ultimate loss of the student denotes a blend of the soft and hard target losses:

(8) $$\begin{array}{c} L_{\mathrm{SLD}}=\alpha L_{\mathrm{soft}}+\left(1-\alpha \right)L_{\mathrm{hard}}, \end{array}$$

where α serves as a weighting factor that balances the contributions of the soft target loss and the hard target loss. Typically, this parameter is adjusted to place greater emphasis on the soft targets, ensuring that the student effectively involves the subtle information distilled from the teacher model.

### 3.3. Representation Distillation

Through knowledge distillation, the student model can leverage the soft labels generated by the teacher model, which encapsulate the relative relationships between classes to adjust the learning weights based on the knowledge extracted from the teacher model for the purpose of graph anomaly detection. To fully exploit the information in the auxiliary set and ensure that the student model captures more detailed node features and achieves better generalization for few-shot anomaly detection, we present the short and long representation distillation ways. This ensures the effective transfer of information from all graph convolutional layers of the teacher model to the student model, thereby improving the student model’s performance in graph anomaly detection.

**Short representation distillation**. For graph anomaly detection tasks, the homophilic neighbors tend to belong to the same category [47,48]. Hence, we minimize the embedding distance (i.e., maximizing the embedding similarity) of two homophilic adjacent nodes in the student model. To this end, we first identify the homophilic nodes and then build the shot distillation loss. Similarly to [47], we adopt GPNN [49] to detect homophilic nodes. Concretely, we leverage a pointer network to compute attention vectors (scores) and then select the most relevant nodes from neighborhoods according to these scores. Since the attention scores can denote the relevant relationship with a given node [49], we use them to identify homophilic nodes: two nodes are regarded as homophilic if they have higher attention scores than a threshold. The optimal threshold can be determined by the existing training data.

Furthermore, we utilize the knowledge of the teacher to enforce this constraint. To achieve this, we ensure that the local structure between each pair of homophilic nodes (such as the *i*- and *j*-th nodes) in the teacher is maintained in the student. Based on this, we define the short representation distillation loss as follows:

(9) $$\begin{array}{cc} L_{\mathrm{short}}=\sum_{i\in C}\sum_{j\in N^{h}\left(i\right)} & \exp\left(-\frac{1}{\sigma^{2}}{∥\phi_{T}^{i}\left(A,X\right)-\phi_{T}^{j}\left(A,X\right)∥}_{2}^{2}\right)\\ \; & \times {∥\phi_{S}^{i}\left(A,X\right)-\phi_{S}^{j}\left(A,X\right)∥}_{2}^{2}, \end{array}$$

where C denotes the target set, $N^{h}\left(i\right)$ represents the first-order homophilic neighborhood set of the *i*-th node, and A and X refer to the adjacent and attribute matrices, individually. $\phi_{T}^{i}\left(A,X\right)$ and $\phi_{S}^{i}\left(A,X\right)$ denote the representations of the *i*-th node generated by the teacher and student, respectively.

Equation (9) enforces a substantial penalty when the embeddings of two adjacent nodes (i.e., the *i*-th and *j*-th nodes in the teacher model) are positioned too closely in the student model. In essence, minimizing Equation (9) guarantees that, if the *i*-th and *j*-th nodes are homophilic neighbors in the teacher model, they will likewise be adjacent in the student model. This equation enables the transfer of information—particularly the local structure of each node’s embedding across all layers of the graph convolutional network in the teacher model—ensuring that the representations of adjacent nodes in the student model remain similar. As a result, short representation distillation preserves the local structure of the teacher model, effectively guiding the training of the student model.

**Long representation distillation**. Following the local structure preservation achieved through the short representation distillation, we introduce long representation distillation to maintain the global structure, leveraging information from the teacher model. The preservation of both short and long structures offers complementary insights [40,50]. Specifically, we begin by extracting node embeddings in the target set using the teacher. In order to maintain the global structural information, we then compute the mean squared error between each node’s representation in the teacher and its corresponding one in the student. The long representation distillation loss is consequently defined as follows:
  (10) $$\begin{array}{c} L_{\mathrm{long}}={∥\phi_{S}\left(A,X\right)-\phi_{T}\left(A,X\right)∥}_{2}^{2}. \end{array}$$
  Equation (10) leverages the information from each graph convolutional tier in the teacher to maintain the holistic structural information from the target set. Consequently, each node’s embedding in the student model closely matches the one of the corresponding item from the teacher. This holistic structural alignment addresses the challenge posed by the limited availability of training samples (i.e., labeled data on the target set) in the student.
**Representation distillation loss**. We combine the short objective function in Equation (9) with the long objective function in Equation (10) to formulate the final loss for our designed approach:
  (11) $$\begin{array}{c} L_{\mathrm{RDL}}=\left(1-\beta \right)L_{\mathrm{short}}+\beta L_{\mathrm{long}}, \end{array}$$
  where β serves as a superparameter that balances the knowledge derived from both partial and holistic structural information. Equation (11) proposes a couple of distilled losses to maintain the partial and holistic structures within the target data. This method strengthens the resilience of the student model by minimizing the influence of poorly trained nodes while efficiently leveraging information from the auxiliary dataset.

In our developed distillation approach, short representation distillation capitalizes on the similarity of embeddings at each convolution tier within the teacher to preserve the local structural information of the target data. In contrast, long distillation employs the representations of all nodes across every convolution tier in the teacher to maintain the global structural information of the target data.

### 3.4. Overall Objective Function

To effectively leverage both class knowledge and representation information, we introduce a unified loss function that merges the knowledge distillation loss with the representation distillation loss. This integration creates a more powerful distillation framework. The resulting combined loss function is formulated as follows:

(12) $$\begin{array}{c} L=\gamma \cdot L_{\mathrm{SLD}}+\left(1-\gamma \right)\cdot L_{\mathrm{RDL}}, \end{array}$$

where γ stands for a balancing factor to adjust the relative significance of the two loss components. This term guarantees that the student tightly emulates the teacher decision-making process. By employing this loss function, the distillation process not only preserves the classification strengths of the teacher but also enables the student to acquire richer and more comprehensive node representations. Overall, this integrated loss function effectively harnesses both knowledge and representation distillation, facilitating notable performance enhancements in the student model, even with limited training samples.

For computational efficiency, the graph convolution operation in the pre-training phase, based on GATs, has a per-layer time complexity of O(L·(E+V)), where *V* and *E* are the number of nodes and edges, and *L* is the number of layers. During the soft label distillation phase, the cross-entropy loss computation for the teacher and student models has a complexity of O(n), where *n* is the number of samples. In the representation distillation phase, both short and long representation distillation methods have a complexity of O(V·d), as they involve embedding comparisons between neighboring nodes or between teacher and student models, where *d* is the embedding dimension. The overall objective function combines the soft label distillation loss (LSLD) and representation distillation loss (LRDL), resulting in a total time complexity of O(L·(E+V)+n+V·d). In practice, the dominant factors are the graph size (*E*, *V*) and embedding dimension *d*.

## 4. Experiment

In this section, we perform empirical evaluations to demonstrate the effectiveness of the proposed DualKD. We mainly investigate the efficacy of the proposed model, ablation study and the role of auxiliary network number.

### 4.1. Experimental Setup

**Datasets**. We conduct experiments on two types of datasets: Ground-truth anomaly graphs: Amazon is a co-purchase network and Yelp is a transaction network, both of which have ground-truth labels of the anomalies; injected anomaly graphs: PubMed and Reddit are two citation networks, with injected anomaly labels. Attribute and structural anomalies are injected into these two datasets using the injection methods of previous studies [15,51]. Table 1 summarizes the statistics of each dataset.

- **Amazon** [16] is collected from Amazon.com and contains product reviews across various categories. The reviewers are classified into two classes, abnormal (reviewers with suspicious review patterns) and normal (reviewers with regular review patterns) according to the Amazon anti-fraud detection algorithm. We select products in the same category to construct each network, where nodes represent reviewers and there is a link between two reviewers if they have reviewed the same product. We apply the bag-of-words model on top of the textual contents to obtain the attributes of each node.
- **Yelp** [52] is collected from Yelp.com and contains reviews for restaurants in several states of the U.S. where the restaurants are organized by ZIP codes. The reviewers are classified into two classes, abnormal (reviewers with only filtered reviews) and normal (reviewers with no filtered reviews) according to the Yelp anti-fraud filtering algorithm. We select restaurants in the same location according to ZIP codes to construct each network where nodes represent reviewers and there is a link between two reviewers if they reviewed the same restaurant. We apply the bag-of-words model on top of the textual contents to obtain the attributes of each node.
- **PubMed** [53] is a citation network where nodes represent scientific articles related to diabetes and edges are citations relations. Node attribute is represented by a TF/IDF-weighted word vector from a dictionary which consists of 500 unique words. We randomly partition the large network into non-overlapping sub-networks of similar size.
- **Reddit** [54] is collected from an online discussion forum where nodes represent threads and an edge exits between two threads if they are commented on by the same user. The node attributes are constructed using the averaged word embedding vectors of the threads. Similarly, we extract non-overlapping sub-networks from the original large network for our experiments.

Note that, except the Amazon and Yelp dataset, we are not able to access ground-truth anomalies for PubMed and Reddit. Thus, following the works [15,34], we refer to two anomaly injection methods [55,56] to inject a combined set of anomalies (i.e., structural anomalies and attribute anomalies) by perturbing the topological structure and node attributes of the original network, respectively. To inject structural anomalies, we adopt the approach used by [55] to generate a set of small cliques since a small clique is a typical abnormal substructure in which a small set of nodes are much more closely linked to each other than average [57]. Accordingly, we randomly select *c* nodes (i.e., clique size) in the network and then make these nodes fully linked to each other. By repeating this process *K* times (i.e., *K* cliques), we can obtain K×c structural anomalies. In our experiment, we set the clique size *c* to 15. In addition, we leverage the method introduced by [56] to generate attribute anomalies. Specifically, we first randomly select a node *i* and then randomly sample another 50 nodes from the network. We choose the node *j* whose attributes have the largest Euclidean distance from node *i* among the 50 nodes. The attributes of node *i* will then be replaced with the attributes of node *j*.

**Baselines**. We assess the identification results of our DualKD against the following baselines:
  - **Autoencoder** [58] is an unsupervised deep autoencoder model which introduces an anomaly regularizing penalty based upon L1 or L2 norms.
  - **Radar** [59] is an unsupervised method that detects anomalies on an attributed network by characterizing the residuals of attribute information and its coherence with network structure.
  - **DOMINANT** [15] is a GCN-based autoencoder framework which computes anomaly scores using the reconstruction errors from both network structure and node attributes.
  - **DeepSAD** [22] is a deep learning approach for general semi-supervised anomaly detection. In our experiment, we leverage the node attribute as the input feature.
  - **SemiGNN** [28] is a semi-supervised GNN model, which leverages the hierarchical attention mechanism to better correlate different neighbors and different views.
  - **BWGNN** [31] is a method equipped with spectral and spatial localized band-pass filters that can better address the right-shift phenomenon in graph anomalies.
  - **Meta-GDN** [21] is a new family of graph neural network that not only leverages a small number of labeled anomalies to enforce statistically significant deviations between abnormal and normal nodes on a network, but also incorporates a cross-network meta-learning algorithm to enable few-shot network anomaly detection.
  - **CAGAD** [47] is a data augmentation-based method for graph anomaly detection that can produce counterfactual augmented data to enhance detection performance.
**Experiment setting**. For each dataset, we extract 5 networks, among which 4 networks are considered as the auxiliary networks and 1 for the target network. For each one, we adopt 10 labeled anomalous nodes for model training. We use the following metrics to conduct a comprehensive evaluation of the performance of different anomaly detection methods:
  - **AUC-ROC** is widely used in previous anomaly detection research [15,59]. Area under the curve (AUC) is interpreted as the probability that a randomly chosen anomaly receives a higher score than a randomly chosen normal object.
  - **AUC-PR** is the area under the curve of precision against recall at different thresholds, and it only evaluates the performance on the positive class (i.e., abnormal objects). AUC-PR is computed as the average precision as defined in [60,61].
  - **Precision@K** is defined as the proportion of true anomalies in a ranked list of *K* (e.g., 10) objects. We obtain the ranking list in descending order according to the anomaly scores that are computed from a specific anomaly detection algorithm.

### 4.2. Overall Detection Performance

In the experiments, we evaluate the performance of the proposed DualKD by comparing with the included baseline methods. We present the evaluation results with respect to AUC-ROC and AUC-PR in Table 2. Accordingly, we have the following observations: (1) In terms of AUC-ROC and AUC-PR, our approach DualKD outperforms all the other compared methods by a significant margin. This indicates that our method can effectively distill knowledge from the teacher model to the student and improve detection performance. (2) Unsupervised methods (e.g., DOMINANT, Radar) are not able to leverage supervised knowledge of labeled anomalies and therefore have limited performance. Semi-supervised methods (e.g., DeepSAD, SemiGNN) also fail to deliver satisfactory results. The possible explanation is that they require a relatively large number of labeled data, which makes them less effective in our evaluation.

### 4.3. Ablation Study

Here, we examine the influence of various components in our designed model. There are mainly two components in DualKD: the soft label distillation and the representation distillation. Hence, we consider the following variants: (1) *DualKD-Base* is the balanced backbone model, which is the basic learning framework that removes the soft label distillation and the representation distillation. (2) *DualKD-Soft* is the learning framework incorporating the soft label distillation. (3) *DualKD-Repr* is the learning framework with the representation distillation. (4) *DualKD-Full* is our proposed model fully involving these components.

**Figure 2:**
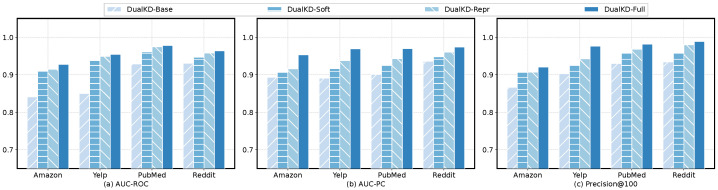
Detection performance of DualKD and its variants.

The experimental results on the four datasets are presented in Figure 2. We summarize the observations from this figure as follows. First, *DualKD-Full* performs the best while *DualKD-Base* performs the worst, suggesting that the main components we designed can substantially enhance the detection results. Second, *DualKD-Soft* outperforms *DualKD-Base* by a certain margin, which validates the effectiveness of the proposed DualKD in completing knowledge transfer for node anomaly detection. Third, *DualKD-Repr* outperforms *DualKD-Soft*, which is primarily because the balanced backbone network with representation distillation in DualKD is able to effectively utilize node information and learn highly expressive node representations. These suggest that our designed method can efficiently distill the information from the auxiliary data to the target data.

### 4.4. Role of Labeled Samples

In order to verify the effectiveness of DualKD in few-shot as well as one-shot network anomaly detection, we evaluate the performance of DualKD with different numbers of labeled anomalies on the target network (i.e., 1-shot, 3-shot, 5-shot, and 10-shot). For these settings, we adjust the batch size to 2, 4, 8, and 16, respectively. Also, we keep the number of labeled anomalies on auxiliary networks as 10. Table 3 summarizes the AUC-ROC/AUC-PR performance of DualKD under different few-shot settings. By comparing the results in Table 2 and Table 3, we can see that even with only one labeled anomaly on the target network (i.e., one-shot), DualKD can still achieve good performance and outperform the baseline methods. In the meantime, we can clearly observe that the performance of DualKD increases with the growth of the number of labeled anomalies, which demonstrates that DualKD can be better fine-tuned on the target network with more labeled examples.

### 4.5. Sensitivity and Robustness Analysis

**Figure 3:**
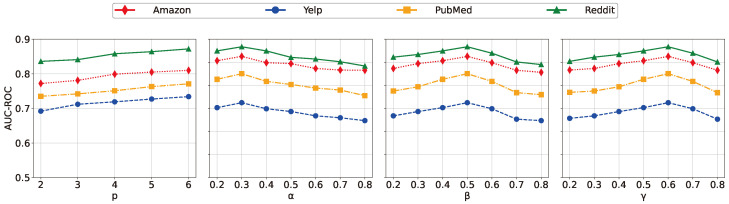
Sensitivity analysis for the number of auxiliary networks *P* and the weight α, β, and γ.

In this section, we further analyze the sensitivity and robustness of the proposed DualKD. By providing different numbers of auxiliary networks during training, the model sensitivity results with respect to AUC-ROC are presented in Figure 3. Specifically, we can clearly find that (1) as the number of auxiliary networks increases, DualKD achieves constantly stronger performance on the four datasets. It shows that more auxiliary networks can provide better knowledge during the training process, which is consistent with our intuition; (2) DualKD can still achieve a relatively good performance when training with a small number of auxiliary networks (e.g., *p* = 2), which demonstrates the strong capability of its base model. For instance, on the Yelp data, the AUC-ROC performance only decreases by 0.042 when the amount of auxiliary networks is reduced from p=6 to p=2.

The parameters α, β, and γ are used to balance the importance of the soft and hard target losses, the short and long objective information, and the soft label and the representation distillation losses, individually. To examine their impact, we analyze different values of α, β, and γ, and present the results in Figure 3. The data show that model performance gradually improves as γ increases from a lower value, followed by a relatively sharp decline as γ continues to rise. This trend suggests that, initially, a higher γ enhances the influence of the knowledge distillation loss ($L_{\mathrm{SLD}}$), enabling the student model to better replicate the decision-making process of the teacher model, thus boosting overall performance. However, as γ becomes too large, the representation distillation loss ($L_{\mathrm{RDL}}$) is de-emphasized, which may hinder the model’s ability to effectively learn node representations, leading to a decrease in performance. It is evident that knowledge distillation progressively contributes to performance enhancement, but if its weight is too dominant, it can significantly impair the model’s representation learning capabilities. The peak performance is observed at γ=0.6, indicating that this balance point between knowledge and representation distillation enables the model to achieve optimal results. Additionally, the results for α and β exhibit similar trends, indicating that properly considering the information, the soft and hard target losses, and the short and long objective information, is crucial to obtain better performance.

## 5. Conclusions

In this paper, we proposed a dual-level knowledge distillation-based graph anomaly detection framework, DualKD, to address the challenge of few-shot anomaly detection in graphs. We introduced a soft label loss which adopts the teacher model to generate soft labels that encapsulate the relative relationships between normal and anomalous nodes. We designed a representation distillation approach including both short and long representation distillation, which ensures the effective transfer of information from all graph convolutional layers of the teacher model to the student model, improving the robustness and performance of the latter. Through extensive experiments, we demonstrated the effectiveness of DualKD in utilizing the knowledge from the teacher model to enhance the detection performance in few-shot scenarios.

## Figures and Tables

**Table 1:** Statistics of evaluation datasets. $r_{1}$ denotes the ratio of labeled anomalies to the total anomalies and $r_{2}$ is the ratio of labeled anomalies to the total number of nodes.

Datasets	Amazon	Yelp	PubMed	Reddit
# nodes	3200	4872	3675	15,860
# edges	29,000	43,728	8895	136,781
# features	8000	10,000	500	602
# anomalies	160	223	201	796
$r_{1}$ (avg.)	5.00%	4.48%	4.97%	1.26%
$r_{2}$ (avg.)	0.25%	0.21%	0.27%	0.063%

**Table 2:** Performance comparison results with respect to AUC-ROC, AUC-PR, and Precision@K on four datasets.

Methods	Amazon			Yelp			PubMed			Reddit		
	A-ROC	A-PR	P@K	A-ROC	A-PR	P@K	A-ROC	A-PR	P@K	A-ROC	A-PR	P@K
Autoencoder	0.4211	0.0539	0.4125	0.3756	0.0423	0.3874	0.5755	0.1873	0.5678	0.5183	0.0712	0.5027
Radar	0.4814	0.0753	0.4547	0.4602	0.0623	0.4929	0.5286	0.1156	0.5891	0.5039	0.0665	0.4614
DOMINANT	0.4507	0.0683	0.4421	0.3657	0.0412	0.5859	0.5842	0.2365	0.4896	0.7223	0.3477	0.6234
DeepSAD	0.3987	0.0575	0.3674	0.3973	0.0465	0.4057	0.4213	0.0483	0.4295	0.2987	0.0483	0.2561
SemiGNN	0.4726	0.0635	0.4489	0.4023	0.0414	0.5117	0.4583	0.0352	0.4645	0.5518	0.0855	0.5223
Meta-GDN	0.5026	0.0654	0.5549	0.4978	0.0585	0.5542	0.5238	0.3066	0.5317	0.5196	0.1349	0.5749
BWGNN	0.5106	0.0788	0.6554	0.5784	0.1095	0.5574	0.6497	0.3379	0.6186	0.7357	0.3572	0.7129
CAGAD	0.7894	0.1213	0.7823	0.6783	0.1327	0.6557	0.7367	0.4386	0.6534	0.8113	0.3794	0.7412
**DualKD**	**0.8103**	**0.1394**	**0.7998**	**0.7246**	**0.1757**	**0.6745**	**0.7614**	**0.4854**	**0.6543**	**0.8987**	**0.3959**	**0.7649**

**Table 3:** Few-shot performance evaluation of DualKD with respect to AUC-ROC, AUC-PR, and Precision@K on four datasets.

Setting	Amazon			Yelp			PubMed			Reddit		
	A-ROC	A-PR	P@K	A-ROC	A-PR	P@K	A-ROC	A-PR	P@K	A-ROC	A-PR	P@K
1-shot	0.7810	0.1071	0.7784	0.7022	0.1569	0.7123	0.7421	0.4623	0.7731	0.8208	0.3801	0.7954
3-shot	0.7991	0.1193	0.7915	0.7094	0.1642	0.7375	0.7483	0.4681	0.7845	0.8289	0.3867	0.8026
5-shot	0.8056	0.1269	0.7842	0.7173	0.1694	0.7537	0.7537	0.4746	0.8092	0.8342	0.3897	0.8283
10-shot	0.8103	0.1394	0.7998	0.7246	0.1757	0.7745	0.7614	0.4854	0.7843	0.8426	0.3959	0.8949

## Data Availability

Data available upon request due to restrictions, e.g., privacy or ethical. The data presented in this study are available upon request from the corresponding author.
